# Um Novo Preditor de Risco no Infarto Agudo do Miocárdio. Ainda tem Lugar para Mais Um?

**DOI:** 10.36660/abc.20220367

**Published:** 2022-07-07

**Authors:** Dalton Bertolim Précoma

**Affiliations:** 1 Angelina Caron Hospital Society Campina Grande do Sul PR Brasil Angelina Caron Hospital Society, Campina Grande do Sul, PR – Brasil; 2 Sociedade Hospitalar Angelina Caron – Cardiologia Campina Grande do Sul PR Brasil Sociedade Hospitalar Angelina Caron – Cardiologia, Campina Grande do Sul, PR – Brasil

**Keywords:** Doenças Cardiovasculares, Fatores de Risco, Infarto do Miocárdio/complicações, Aterosclerose, Mediadores da Inflamação, Placa Aterosclerótica, Colesterol

A cardiologia é uma das especialidades que tradicionalmente utiliza a evidência científica na prática diária, tanto na estratificação de risco como no diagnóstico, na terapêutica e no prognóstico. Um dos temas mais discutidos neste sentido, é a aterosclerose e a inflamação, despertando um grande interesse pelo contínuo conhecimento adquirido ao longo dos últimos dois séculos. Vários autores se destacam neste contexto histórico, por exemplo, Rudolf Virchow no século 19 que descreveu a associação da aterosclerose com a inflamação; Marchand em 1904 que sugeriu a relação entre a aterosclerose e o processo de obstrução das artérias e em 1908, Ignatowski, que observou a relação entre o colesterol da dieta e a aterosclerose. Ao longo destes últimos 100 anos, uma série de artigos elucidaram a sequência fisiopatogênica que hoje conhecemos. O entendimento da formação e a evolução da placa aterosclerótica, através de complexos mecanismos moleculares e da imunidade inata e adaptativa, que culminam com o infarto agudo do miocárdio (IAM) estão bem estabelecidos.^1–4^ Em 1974, Friedman GD et al., descreveram o papel da contagem dos leucócitos no prognóstico do IAM,^5^ e na sequência, outros estudos destacaram a importância destas células no processo de deterioração e recuperação do miocárdio infartado.^6–8^ Nesta mesma linha de investigação, Coste MER et al., investigaram as citocinas em pacientes com infarto do miocárdio com supra de ST (IAMCSST) e a relação com a função ventricular. Observaram um balanço das citocinas pró-inflamatórias e anti-inflamatórias, exceto da IL-6, sugerindo um risco inflamatório residual.^9^

Além destes aspectos relacionados com a placa aterosclerótica e a atividade inflamatória no IAM, outro processo relacionado com estas células chamadas imuno-inflamatórias, como as plaquetas, os leucócitos, os neutrófilos e os linfócitos, ganharam destaque inicialmente na área da oncologia, ao ser descritos como um marcador prognóstico confiável na progressão de vários tumores malignos, pelo chamado “índice de imuno-inflamação sistêmica” (IIIS). A revisão sistemática e metanálise de Zhong et al.,^10^ ressalta a importância deste índice na predição de sobrevida, sendo que elevadas taxas foram associadas a um pior prognóstico em tumores sólidos.^10^ Além das neoplasias, outros fatores alteram a IIIS, tais como a idade, obesidade, diabete tipo 2, estresse emocional, esteroides exógenos, hormônios sexuais endógenos, distúrbios hematológicos, acidente vascular cerebral, embolia pulmonar e trauma.^11^

As células brancas, como os leucócitos e neutrófilos, são abundantes e as primeiras a atuarem como pró-inflamatórios na área infartada. Já as plaquetas, participam do processo pró-inflamatório e pró-trombótico, além de outras ações a longo prazo na aterosclerose. Os linfócitos são células com características imunes cuja ação anti-inflamatória promove a proteção e a recuperação do tecido infartado ou as células já deterioradas. Por estas funções celulares principalmente na fase aguda do infarto, nos últimos anos, muitos autores relataram estes elementos celulares como valor prognóstico nas síndromes coronarianas agudas (SCA). Takahashi et al. em 2007 estudaram 116 casos de IAM da parede anterior, dentro das 12 primeiras horas, submetidos a angioplastia primária, com o objetivo de verificar o grau de acometimento microvascular do ventrículo esquerdo (VE). A análise multivariada demonstrou que o grau dos neutrófilos foi um preditor independente do acometimento microvascular após a angioplastia.^12^

Além da importância destes índices prognósticos, recentemente alguns estudos demonstraram o papel da terapêutica anti-inflamatória na redução de desfechos cardiovasculares. Como o estudo *The Canakinumab Anti-Inflammatory Thrombosis Outcomes Study (CANTOS)* que demonstrou a redução dos desfechos maiores pelo tratamento anti-inflamatório com esta substância monoclonal com ação na interleucina-1 Beta, diminuindo níveis da proteína C reativa ultrassensível.^13^ Outro importante estudo, *Colchicine Cardiovascular Outcomes Trial (*COLCOT) demonstrou a redução de 23% dos desfechos maiores com a utilização da colchicina na doença coronariana crônica.^14^

Num estudo analisando estes três elementos celulares pela relação entre as plaquetas (P), os neutrófilos (N) e os linfócitos (L) - (IIIS: PxN/L), Yang et al. descreveram este índice como preditor de risco independente, sendo superior aos fatores de risco tradicionais.^15^

Nesta edição dos Arquivos Brasileiros de Cardiologia, Saylik e Akbulut,^16^ relacionam o IIIS, utilizando mesmo critério de Yang et al., estudando 843 pacientes portadores IAMCSST, submetidos a angioplastia primária. O índice elevado, foi associado com a idade avançada, maiores taxas de mortalidade cardiovascular, infarto do miocárdio não fatal, acidente vascular cerebral não fatal, hospitalização por insuficiência cardíaca, revascularização miocárdica e eventos cardiovasculares maiores. Eles concluíram que o IIIS é um preditor independente. Este estudo ressalta que a utilização da relação das plaquetas, neutrófilos e linfócitos, é superior na predição de risco, do que a razão do neutrófilo-linfócito e à razão plaqueta-linfócito, utilizadas em outros estudos.^17,18^ Isto se deve provavelmente à associação de mecanismos compostos da resposta imuno-inflamatória na resposta da agressão principalmente pelos neutrófilos nos primeiros dias e da regeneração celular pela resposta imune e apoptótica dos linfócitos na sequência.^19^

Devemos considerar alguns aspectos metodológicos, pois o estudo foi retrospectivo, unicêntrico, sendo o acompanhamento por telefone ou registros hospitalares e certificados de óbito para a causa de morte. Além disso, não foram relatadas as medicações prévias utilizadas, como as estatinas, colchicina, corticoides, quimioterápicos e outras que possam influenciar nos resultados. Outro fato que poderia interferir na análise dos resultados, foi a coleta dos dados de mortalidade por telefone, prontuários e certificados de óbito. Porém, com os resultados baseados em exames de entrada do hospital e no método empregado, o estudo permitiu demonstrar que este índice de imuno-inflamação tem muita importância prognóstica, sendo facilmente incorporado na prática diária devido a seu baixo custo e facilidade de acesso.

Concluímos que o tema nos remete a “mais uma” ferramenta de estratificação de risco do infarto do miocárdio, de maneira prática e de baixo custo, que facilmente pode ser incorporada à nossa prática.
