# Supplementary material for: Um Novo Preditor de Risco no Infarto Agudo do Miocárdio. Ainda tem Lugar para Mais Um?
Source: Arq Bras Cardiol. 2022 Jul 7;119(1):23–4. [Article in Portuguese] doi: 10.36660/abc.20220367 (PMC9352120; doi:10.36660/abc.20220367)
Supplement: Translation

# Translation: A New Risk Predictor in Acute Myocardial Infarction. Is There Still Room for One More?

**Authors:** Dalton Bertolim Précoma

**Affiliations:** 1 Campina Grande do Sul PR Brazil Angelina Caron Hospital Society, Campina Grande do Sul, PR – Brazil; 2 Campina Grande do Sul PR Brazil Sociedade Hospitalar Angelina Caron – Cardiologia, Campina Grande do Sul, PR – Brazil

**Keywords:** Cardiovascular Diseases, Risk Factors, Myocardial Infarction/complications, Atherosclerosis, Inflammation Mediators, Plaque, Atherosclertic, Cholesterol

Cardiology is one of the specialties that traditionally uses scientific evidence in daily practice, both in risk stratification and diagnosis, therapy and prognosis. One of the most discussed topics is atherosclerosis and inflammation, arousing great interest in the continuous knowledge acquired over the last two centuries. Several authors stand out in this historical context, for example, Rudolf Virchow in the 19th century who described the association of atherosclerosis with inflammation; Marchand in 1904, who suggested the relationship between atherosclerosis and the process of clogging of the arteries and in 1908, Ignatowski who observed the relationship between dietary cholesterol and atherosclerosis. Over the last 100 years, many articles have elucidated the pathophysiological sequence that we know today. Understanding atherosclerotic plaque formation and evolution through complex molecular mechanisms and innate and adaptive immunity, which culminate in acute myocardial infarction (AMI), is well established.^1–4^ In 1974, Friedman GD et al., described the role of leukocyte count in the prognosis of AMI,^5^ and subsequently, other studies highlighted the importance of these cells in the deterioration and recovery of infarcted myocardium.^6–8^ In the same line of investigation, Coste MER et al. investigated cytokines in patients with ST-segment elevation myocardial infarction (STEMI) and the relationship with ventricular function. They observed a balance of pro-inflammatory and anti-inflammatory cytokines, except for IL-6, suggesting a residual inflammatory risk.^9^

In addition to these aspects related to atherosclerotic plaque and inflammatory activity in AMI, another process related to these so-called immuno-inflammatory cells, such as platelets, leukocytes, neutrophils, and lymphocytes, initially gained prominence in the field of oncology when described as a reliable prognostic marker in the progression of various malignant tumors, by the so-called “systemic immuno-inflammation index” (IIIS). The systematic review and meta-analysis by Zhong et al.^10^ emphasize the importance of this index in predicting survival since high rates were associated with a worse prognosis in solid tumors.^10^ In addition to neoplasms, other factors alter IIIS, such as age, obesity, type 2 diabetes, emotional stress, exogenous steroids, endogenous sex hormones, hematological disorders, stroke, pulmonary embolism, and trauma.^11^

White cells, such as leukocytes and neutrophils, are abundant and the first to act as pro-inflammatory in the infarcted area. On the other hand, platelets participate in the pro-inflammatory and prothrombotic processes and other long-term actions in atherosclerosis. Lymphocytes are cells with immune characteristics whose anti-inflammatory action promotes the protection and recovery of infarcted tissue or cells that have already deteriorated. Due to these cellular functions, mainly in the acute phase of infarction, in recent years, many authors have reported these cellular elements as a prognostic value in acute coronary syndromes (ACS). Takahashi et al. in 2007, studied 116 cases of anterior wall AMI, within the first 12 hours, submitted to primary angioplasty to verify the degree of microvascular involvement of the left ventricle (LV). Multivariate analysis showed that neutrophil grade was an independent predictor of microvascular involvement after angioplasty.^12^

In addition to the importance of these prognostic indices, recently, some studies have demonstrated the role of anti-inflammatory therapy in reducing cardiovascular outcomes. The Canakinumab Anti-Inflammatory Thrombosis Outcomes Study (CANTOS) demonstrated the reduction of major outcomes by anti-inflammatory treatment with this monoclonal substance with action on interleukin-1 Beta, decreasing levels of ultrasensitive C-reactive protein.^13^ Another important study, the Colchicine Cardiovascular Outcomes Trial (COLCOT), demonstrated a 23% reduction in major outcomes using colchicine in chronic coronary heart disease.^14^

In a study analyzing these three cellular elements by the relationship between platelets (P), neutrophils (N) and lymphocytes (L) - (IIIS: PxN/L), Yang et al. described this index as an independent risk predictor, being superior to traditional risk factors.^15^

In this issue of *Arquivos Brasileiros de Cardiologia*, Saylik and Akbulut,^16^ list the IIIS, using the same criteria as Yang et al., studying 843 patients with STEMI who underwent primary angioplasty. The high index was associated with older age, higher cardiovascular mortality rates, non-fatal myocardial infarction, non-fatal stroke, hospitalization for heart failure, myocardial revascularization, and major cardiovascular events. They concluded that IIIS is an independent predictor. This study emphasizes that the use of the ratio of platelets, neutrophils and lymphocytes is superior in predicting risk than the neutrophil-lymphocyte ratio and the platelet-lymphocyte ratio used in other studies.^17,18^ This is probably due to the combination of mechanisms composed of the immuno-inflammatory response in response to aggression mainly by neutrophils in the first days and cell regeneration by the immune and apoptotic response of lymphocytes in the sequence.^19^

We must consider some methodological aspects, as the study was retrospective, single center, with follow-up by telephone or hospital records and death certificates for the cause of death. In addition, previous medications used, such as statins, colchicine, steroids, chemotherapy drugs and others that could influence the results, were not reported. Another fact that could interfere in the analysis of the results was the collection of mortality data by telephone, medical records and death certificates. However, with the results based on hospital entrance exams and the method used, the study showed that this index of immuno-inflammation has great prognostic importance, being easily incorporated into daily practice due to its low cost and ease of access.

We conclude that the topic reminds us of “one more” tool for stratifying the risk of myocardial infarction in a practical and low-cost way, which can easily be incorporated into our practice.
